# Supplementary material for: MetaRibo-Seq measures translation in microbiomes
Source: Nat Commun. 2020 Jun 29;11:3268. doi: 10.1038/s41467-020-17081-z (PMC7324362; doi:10.1038/s41467-020-17081-z)
Supplement: Supplementary file 10 — Supplementary Data 7 [file 41467_2020_17081_MOESM10_ESM.zip › File2/Confidence_VeryHigh_Taxonomy/110324_out.krona.html]

Javascript must be enabled to view this page.

members
magnitude
magnitudeUnassigned
count
unassigned
taxon
rank

110324\_out

4

2
superkingdom
4

1239
4
phylum

186801
4
class


SRS017916\_contig\_number\_14467
2044939
species
1

order
3
186802

424536
family
3

270497
3
genus

species
3

SRS013965\_contig\_number\_contig-100\_104.210357SRS049959\_contig\_number\_10949SRS144537\_contig\_number\_18807
1946247
